# Diversity of Hemodynamic Reactive Profiles across Persons—Psychosocial Implications for Personalized Medicine

**DOI:** 10.3390/jcm11133869

**Published:** 2022-07-04

**Authors:** Miguel Ángel Gandarillas, Nandu Goswami

**Affiliations:** 1 Department of Social, Work, and Differential Psychology, School of Psychology, Complutense University of Madrid, Campus de Somosagua, Ctra. de Húmera, s/n, Pozuelo de Alarcón, 28223 Madrid, Spain; 2 Physiology Division, Otto Loewi Center of Vascular Biology, Immunity and Inflammation, Medical University of Graz, 8036 Graz, Austria; nandu.goswami@medunigraz.at; 3 Mohammed Bin Rashid University of Medicine and Health Sciences, Dubai P.O. Box 505055, United Arab Emirates

**Keywords:** autonomic nervous system, hemodynamics, predictive health, personalized medicine, psychosocial factors, integrative medicine

## Abstract

This study analyzed the individual differences in hemodynamic time patterns and reactivity to cognitive and emotional tasks, and explored the diversity of psycho-physiological profiles that could be used for the personalized prediction of different diseases. An analysis of heart rate (HR)—blood pressure (BP) relationship patterns across time using cross-correlations (CCs) during a logical-mathematical task and a task recalling negative emotions (rumination) was carried out in a laboratory setting on 45 participants. The results showed maximum HR–BP CCs during the mathematical task significantly more positive than the maximum HR–BP CCs during the rumination task. Furthermore, our results showed a large variety of hemodynamic reactivity profiles across the participants, even when carrying out the same tasks. The most frequent type showed positive HR–BP CCs under cognitive activity, and several positive–negative HR–BP CCs cycles under negative emotional activity. In general terms, our results supported the main hypothesis. We observed some distinct time-based “coordination strategies” in the reactivity of the autonomic nervous system under emotional vs. cognitive loading. Overall, large individual, as well as situational, specificities in hemodynamic reactivity time patterns were seen. The possible relationships between this variety of profiles and different psychosocial characteristics, and the potential for integrative predictive health within the provision of highly personalized medicine, are discussed.

## 1. Introduction

Different general patterns of the Autonomic Nervous System (ANS) are well known to be linked to diseases, such as hypertension [1,2,3,4], depression [5,6,7,8], coronary heart disease [6], as well as to psychological and social stressors [9,10,11,12,13]. Moreover, a body of studies reported on the relationships between psychological/social variables and ANS activity/hemodynamic profiles (e.g., [9,14,15,16]). There is a growing trend to study time patterns related to the ANS, facilitated by different computer-based methods through correlational, dynamic modeling and machine-learning analyses, such as autoregressive integrated moving average (ARIMA), Hidden Markov Models (HMM), artificial neural networks, support vector machine methods, fuzzy methods and rough set theory, factor analyses, and cross-correlations (e.g., [9,10,14,17,18,19,20,21,22,23,24,25,26,27,28]). These computer-based methods allow for a more comprehensive and integrative understanding of the way the ANS behaves as it reacts to different psychological and social factors. 

As the ANS behaves as a complex system, an integrative approach to fully understand the behavior of this system involves analyzing the way the different parts of the system relate and coordinate with each other in their responses to the psychosocial context. Examples in this direction are the studies of the differences in the hemodynamic responses across individuals [8,10,11,13,29], related to psychological traits [10,30] and socio-cultural factors [9,14,15,31,32,33,34,35,36,37,38], grounded in a relevant theoretical basis [34,35,39,40,41,42,43,44,45,46,47,48,49,50,51], and empirical findings on the individual differences in autonomic reactivity [8,31,52,53,54]. Furthermore, we may infer from these studies that it seems more precise to predict ‘autonomic coordination’ patterns (understood here as the common action of the autonomic organs in response to a given situation) than the activity of single variables, such as heart rate, blood pressure, skin conductance, and respiratory measures [53]. A substantiation of such a complex assessment of ANS is the differentiation of two main prototypes of ‘autonomic coordination’ strategies among autonomic variables across time and across individuals. These two prototypes are grounded by theoretical approaches, such as the polyvagal theory [35,55], stating two distinct branches of the parasympathetic nervous system (PNS), one phylogenetically older branch (more related to response to threatening situations) originating in the dorsal motor nucleus, and a newer branch (more related to social life) originating in the nucleus ambiguus. Complementary to this theory, Gray’s [45,46] model defines two systems: the Behavioral Inhibition System (BIS) (more related to sensitivity to punishment and avoidance) and the Behavioral Activation System (BAS) (more related to sensitivity to reward and approach motivation). 

There is a need to further research in this promising direction, as this could be useful for providing better detection, prediction, and prevention of different diseases, such as hypertension and coronary artery disease, towards a more precise personalized and predictive health. Thus, the main goal of the present study was the identification and analysis of the different time patterns of the hemodynamic responses to psychological stimuli, comparing the reactions to cognitive vs. emotional tasks (mathematical vs. rumination on a negative past event, respectively), using the cross-correlation technique. An additional goal was to explore if individual responses may be classified into different types of hemodynamic patterns, suggesting the possibility to find different ‘autonomic coordination’ profiles across groups of individuals Here we suggest that ‘autonomic coordination’ profiles across kinds of tasks and individuals may be placed within a dimension with two poles: On one side, a ‘peripheral’ pattern type, related to the older phylogenetical PNS branch of the polyvagal theory and the Gray’s BIS; and on the other side, a ‘central’ pattern type, more influenced by inputs from the cortical and limbic systems, and related to the newer PNS of the polyvagal theory and the Gray´s BAS. Both of the patterns may be reflected in the heart rate—blood pressure time relationship (HR–BP) using cross-correlations (CCs, that is, correlations between the HR and BP across time, either simultaneously or in different lags). This is described in more detail under the Methods section. 

We find a ‘peripheral’ response pattern of the ANS when significant negative HR–BP CCs are seen, with BP “leading” and HR “delayed” and negatively correlated, that is, changing in the opposite direction. In this case, the organism may tend to re-adjust its physiological balance, especially when facing negative emotional inputs. This could occur possibly via the baroreceptor reflex, with the HR changes following the BP changes [23,36,55,56]. We may find a ‘central’ pattern when the results show significant positive CCs, with HR “leading”, reacting first and then BP ”lagging” (delayed) and changing in the same direction (that is, either increasing or decreasing). This pattern of response indicates a tendency to approach positive or challenging stimuli with a greater cortical role for the autonomic system, with the HR reacting faster than BP [23,36,40,52,53,56]. This could also be attributed to a resetting of the baroreceptor reflex [57]. Significant correlations between HR–BP and other autonomic indices (e.g., skin conductance and respiratory measures) and with psychosocial variables, such as emotional attitudes, parents’ care styles in childhood, and family financial status have also been reported [53].

We hypothesized that the maximum positive HR–BP CCs will occur with the cognitive (mathematical) task (reflecting a ‘central’ pattern), whilst the maximum negative HR–BP CCs will be seen when recalling a negative (rumination on a negative past event) emotional task (reflecting a ‘peripheral’ pattern). In addition, we expected to find several types of hemodynamic grouping across the individuals, ranging from those showing very high, significantly positive CCs responses (characterized as ‘central’ individuals) during the mathematical task to those with very high significantly negative CCs (a ‘peripheral’ group) during the ruminative task. We expected to see *individual—*as well as *situational—specificity* in the hemodynamic reactivity across the participants. 

## 2. Materials and Methods

**Study participants.** Participants were 16 men (mean age 35.4 years) and 29 women (mean age 32.6 years), living in Los Angeles (CA, USA) who were preliminarily screened at the University of California, Los Angeles (UCLA) for significant health problems and use of medications that could affect cardiovascular functions. The total number of volunteer participants who completed the study was 45. 

**Apparatus and Physiological Recordings.** The electrocardiogram (ECG) was monitored with a multi-trace recorder (AcqKnowledge: Biopac System, Santa Barbara, CA, USA). Disposable Ag–AgCl electrodes (ConMed Corp., Utica, NY, USA) were affixed at standard thoracic monitor sites (right clavicle and precordial site V6). Beat-to-beat blood pressure was measured noninvasively, using a Finapres Continuous NIBP Monitor (Ohmeda, Madison, WI, USA). Continuous blood pressure readings were obtained via a finger cuff attached to the third finger of the non-dominant hand. Each signal was continuously recorded (2000 samples/s for the ECG and 62.5 samples/s for BP) during baseline, each task, and recovery.

**Procedure.** The experimental protocol consisted of an initial 10-min baseline period, followed by the (rumination and mathematical) 2.5-min tasks. Each task was separated by a 10-min resting period. The session also ended with a 10-min resting period. The order of tasks was counterbalanced across the subjects.

*Computerized Logical-Mathematical (‘math’) Task*. Participants were asked if the conclusion to a randomly generated syllogism (e.g., if a < b and b < c then a < c) was true or false. They responded by choosing the related button on the personal computer. If an answer was entered within the time limit, a window displaying ‘‘Correct answer!’’ or ‘‘Incorrect answer!’’ appeared. If a participant’s response time was delayed, a window displaying ‘‘Too late!’’ appeared. After completion of each syllogism, a new one appeared immediately on the screen. A period of 5 s was allowed for each question. The task lasted 2 min and 30 s.

*Rumination Task*. The task required participants to recall an episode in which they felt intense anger or rage (i.e., being insulted, experiencing abusive or unfair treatment, witnessing others receiving unfair or abusive treatment). The participants were asked to mentally ruminate on the causes and consequences of this episode until the experimenter instructed them to ‘‘stop.’’ The task lasted 2 min and 30 s.

**Data analysis.** The data analysis was carried out by a researcher not involved in the data collection. The laboratory data were visually checked for errors in the peaks detection. In the case of small single errors (one peak recorded with error), the measures were replaced by the means between the preceding and the following peak measures. With large errors (two or more consecutive peaks with errors), the case was rejected. At the end, there remained 42 cases in the math task and 34 cases in the rumination task, and among them, 31 cases had valid data in both the math and rumination task, for further analyses. Then, the data on the heart rate (beats-per-minute), systolic (SBP), diastolic (DBP) and the mean (MBP) blood pressure (mmHg) were obtained. The data for the whole time series for each task and each participant were re-sampled, selecting 1-s samples in order to match the HR and BP units across time, and to increase the robustness of the analyses. The sample size of the time series per individual in each task was 148–149 measures (in seconds).

The means of the baseline, math and rumination task conditions were obtained for each subject and one-way repeated-measures ANOVAs were conducted for the HR, SBP, DBP and MBP means within the three conditions (baseline, math and rumination tasks).

The HR–SBP, HR–DBP, and HR–MBP CCs for the math and rumination tasks were carried out, estimating the correlations up to 30 positive and 30 negative lags (that is, up to 30 s of delays between the HR and BP time measures in the correlations in either direction) for each participant. Here, the lag number depicts the number of time measures (in seconds) that are delayed between HR and BP in the correlation. For example, five positive lags means that HR “leads” and the BP measure “lags” behind (5 s delayed in time) in estimating the correlation. In negative lags, BP “leads” in time, with HR delayed (“lagged”). The number of lags (±30) was selected, as no relevant correlations between HR and BP were found after 30 s. After several preliminary CC tests trying a different number of lags (up to 50 lags), the results revealed that no maximum CCs above 30 lags occurred in any case. The resulting CCs series (±30 lags) for each of the math and rumination tasks, including all of the valid samples, were grouped according to the signs and shapes of the significant CCs (considered significant when CCs at least two times their standard error, with a 95% confidence interval) on the CCs graphics (that is, the curve of the CCs across the positive and negative lags in seconds). Three researchers independently classified the types and then compared their results, finding consensus in their classification. 

The maximum (either positive or negative) significant cross-correlation was selected in each of the math and rumination tasks for each individual. Three separate one-way repeated-measures ANOVAs between the maximum HR–SBP, HR–DBP, and HR–MBP CCs comparing the math and rumination tasks were carried out. The SPSS package was used for the statistical analyses. 

## 3. Results

Table 1 below shows the differences in the means of HR and BP indices between the baseline, math and rumination tasks. Significant differences (*p* < 0.05) were found in the BP indices, with maximum means during the rumination task.

Regarding the analysis on the HR–BP maximum CCs, all those in the math task were more positive than in the rumination task. However, the repeated-measures ANOVA showed significant effects only for HR–SBP (Wilks’ Lambda = 0.85, *F*(1, 33) = 5.86, *p* = 0.021; partial Eta squared = 0.15), with the math task showing higher positive maximum CCs (mean = 0.15) than the rumination task (mean = −0.09).

Table 2 shows the cases (with valid data in both of the tasks) crossing the types of HR–SBP CCs profiles (the types of the curve in the CCs graphics per person and task) on the math task with those on the rumination task. HR–SBP, HR–DBP and HR–MBP CCs in each case behaved with the same shapes in all of the cases and tasks, so the classification of the profiles are valid for the three measures of HR–BP. 

**Figure 1:**
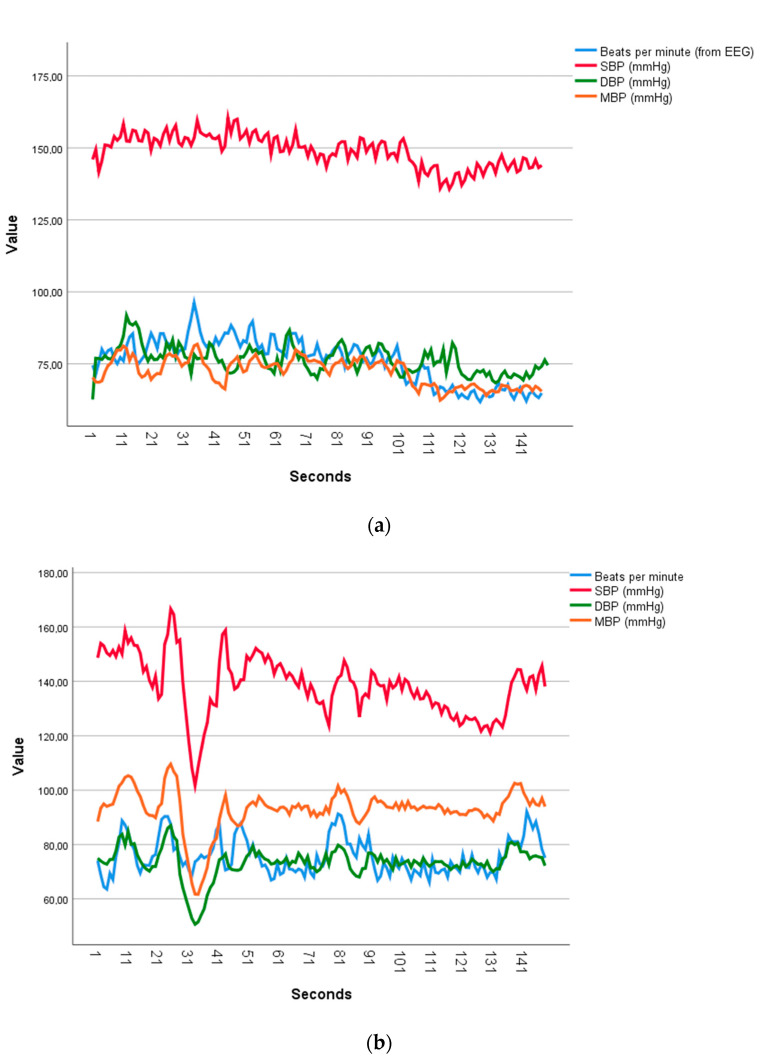
Illustrative case showing the series of HR, SBP, DMB and MBP (in seconds) during (**a**) Math task and (**b**) Rumination task on the same individual of a most frequent type of heart rate–blood pressure cross-correlations (CCs) profile: Cases with significant positive CCs during math task and several cycles of CCs during rumination (95% confidence interval).

The classification of HR–SBP CCs profiles distinguished six types of profiles during the math task (Chi-square (5) = 21.6; *p* = 0.001), being the most frequent types: (1) A majority of significant positive CCs (14 cases out 31); (2) Cases with non-significant CCs (five cases); (3) Cases with two–three positive–negative CC cycles (four cases); and (4) Those with majority of negative CCs (four cases). During the rumination task, there were 6 types of profiles (Chi-square (5) = 17.0; *p* = 0.004), being the most frequent types: (1) Two–three positive-negative CC (several) cycles (13 out of 31); (2) Those showing majority of significant positive CCs (six cases); (3) Those with positive CCs under negative lags and negative CCs under positive lags (six cases); and (4) those with a majority of significantly negative CCs (four cases). A typical example of the most frequent type of individual responses across the math task and the rumination task is shown in Figure 1. 

## 4. Discussion

Our results did not show many significant results in the MANOVAs for the maximum CCs between the mathematical and the rumination tasks, but the trend of higher positive CCs in the mathematical task was unambiguous. However, in the rumination task, the most frequent type showed several CC cycles, which was somewhat discordant to what we had expected.

Several types of hemodynamic profiles were found during the mathematical and rumination tasks. A most frequent tendency reflected a significant positive HR–BP relationship during the logical math task (with HR changing slightly prior to the BP changes), partly supporting the hypothesis. In any case, the results showed a very interesting rich variety of types of individual responses under the same tasks, suggesting an important diversity of profiles of hemodynamic “strategies” across individuals. 

In general terms, our findings support the main assumptions. Namely, the most frequent HR–BP profiles showed a positive HR–BP relationship in the mathematics task, with the heart rate changes occurring prior to the BP changes, which could be explained via a strong central, cortical “mediator” role [55]. On the other hand, under negative affective arousal (the rumination task), the positive–negative CC cycles could show a reciprocal balancing HR–BP reactivity when the negative stimuli were longer than just a few seconds. This does not contradict previous results, as it reflects a strategy to search for physiological balance, a major role of a self-regulated autonomic system (a more ‘peripheral’ reactivity), possibly explained by a higher role of negative feedback mechanisms, such as baroreflex [5,57,58], which did not appear under the mathematical task.

The high diversity of physiological (“coordinated”) reactions among the individuals under the same tasks is highly interesting and opens spaces for new fields of research on predictive and personalized health. Our results suggest a tendency for three main types of individuals on ‘autonomic coordination profiles’, with a group showing a ´central´ type of reaction even during the rumination task (i.e., a major tendency for positive HR–BP CCs), a group of individuals with a more ´peripheral´ type of reaction (showing CC cycles), and other individuals with mixed positive–negative reactions (who might be able to adapt hemodynamic patterns to different situations by increasing the ´central´ or ´peripheral´ phases in a more flexible way). It is worth noting that only one case fell out of these three main categories for each task. This suggests a tendency by the majority of individuals to fall within several prototypes of hemodynamic activity.

Based on the theoretical grounds of this study we suggest possible explanations to these differences in the hemodynamic reaction patterns. ‘Peripheral’ individuals may be able to maintain BP under control better than ‘central’ individuals in negative circumstances, using the balancing “strategy”. ‘Central’ individuals might be more able to pursue a challenging task (mathematical task) keeping the motivation and increasing interest in the task (maintaining the parasympathetic activity and increasing the sympathetic activity), in a parasympathetic–sympathetic co-activation “strategy” [43,44]. The relevance of the differential increase on the BP of the ´central´ individuals may also be explained by a norepinephrine-mediated central role of the sympathetic activation [56].

**Possible psychosocial factors as predictors of a diversity of hemodynamic profiles.** Whereas nowadays a majority of studies on predictive medicine focus on genetic factors, it is also important to analyze deeper the psychosocial factors, found as relevant predictors of the health status (as mentioned above), for an integrative personalized healthcare. The results of this study suggest a taxonomy of hemodynamic prototypes in line with other studies which relate them to different psychosocial and cultural contexts [8,15,32,36,38,39,43,52,53]. These studies suggest the existence of general “autonomic coordination” profiles and physiological prototypes linked to psychological characteristics, which predict the general autonomic patterns better than the single behavior of each organ. The specific links between these prototypes and psychosocial variables were found in a previous study [53], with a ‘central’ type of subjects having a more positive attitude towards rewards and less fearful attitude towards punishment, and with more supportive child-rearing in families of a higher economic status, compared to ‘peripheral’ subjects, with a more defensive psychological profile. A third mixed-type was also found, which may mean more flexible, adapted individuals when reacting to contingencies. The present study also supports these profiles, including the relationship between cognitive/emotional activity and autonomic reactivity.

How could the ANS dynamics develop in order to reach such a variety of physiological reacting patterns to same stimuli? Based on Gray’s [45,46], Porges’ [35], and Cacioppo’s [43,44] models, and previous empirical studies [8,15,32,38,41,52,53], we suggest that ANS evolves through a learning process in the person’s emotional development, mainly through child-rearing practices. In their psycho-physiological development, individuals learn how to adapt themselves to their environmental demands and opportunities, according to the type of environment and to emotional rearing patterns. As we learn to interiorize social reward and satisfaction, we also develop the ability to stimulate an intrinsic motivation with a cortical (‘central’) arousal which leads, at the autonomic level, to a tendency to co-activate the SNS and the PNS facing socially rewarding or challenging stimuli [43,44]. This will have the resulting function of approaching a goal. On the other hand, learning to interiorize punishment will facilitate a stronger role of the older branch of the ANS (vs. cortical input) [35] or the Behavioral Inhibition System [45,46] in the reactions to threatening situations. This will allow more “autonomy” to the ANS, resulting in a higher tendency to reciprocal SNS–PNS activation, bringing a functionally (´peripheral´) defensive and balancing trend. These two autonomic types may be also related to two main active and approaching (related to ‘central’) vs. passive or avoiding (related to ‘peripheral’) coping strategies to social stress, which could contribute an explanation of some of the findings of different studies on the topic (e.g., [2,7,10,15,42]).

**Further studies.** We need further studies with larger samples. An additional study with a group of subjects experiencing actual emotions (not only recalling them) could be especially relevant for further validation of these results. Exploring the differences in hemodynamic time patterns in different ages and sexes will also provide additional theoretical and interesting applied information. Furthermore, including new additional methodologies, besides the traditional linear and non-linear modeling, may get deeper in the understanding and predictive capacity of physiological coordination dynamics. The use of the computer power to process large amounts of data will move us forward in identifying, classifying, and predicting the time patterns of relationships between physiological, genetic, psychological, social variables and their relationship with the prognosis of different diseases, allowing the improvement of differential predictions for each individual in personalized medicine. 

The study of the relationship between physiological disorders and autonomic patterns across individuals, including not only genetic but also different psychosocial features, opens an interesting opportunity to apply successful predictive methodologies to personalized medicine. Predictive methodologies have been effective in other disciplines with a long history and might be good references for predictive medicine, e.g., in meteorology. In local weather forecasting, where atmospheric dynamics are influenced by many geographic variables, linear and non-linear dynamical models are less useful, and statistical methods, including large sets of different variables, are widely used, with increasing accuracy. Examples of these methods are HMM, cluster analysis and multiple regression, neural networks, ARIMA, cross-correlations and analog ensemble [59,60,61,62,63,64]. Meteorologists tend to take advantage of the speedy evolution of computer power to include more and more data from further variables, combining “machine-learning-based” methods with dynamic models, intending to acquire the “whole picture” and to apply it to local forecasting (e.g., “Bayesian Networks” [65]).

Applying the logic of current weather forecasting methods based on ‘analogs’, we may analyze an individual’s physiological time patterns being compared to big databases of other individuals with similar characteristics and with different disorders. Thus, present and past physiological patterns may be compared within and between individuals, improving the early detection and prediction of disease onset and evolution, for greater effective primary, secondary and tertiary prevention. The use of non-intrusive and wearable devices for online and ambulatory monitoring may allow better in-home healthcare and prediction of disorders [66,67,68,69].

The present research points to a fruitful approach, which could be promising when linked to different diseases, as it may help in finding the probabilities of risks of disorders according to different ‘autonomic coordination’ types. As an example, we could hypothesize that under stress, a ‘peripheral type’ of person could be more prone to hypertension, whereas a ‘central’ type of person could be more prone to a myocardial infarction. ‘Autonomic coordination’ patterns may give us information about the probabilities related to physiological crises, in order to prevent them. Hopefully, further studies of the relationship between these profiles and psychosocial features and contexts will allow the generation of large data banks on social, cultural, ecological and health psycho-physiological patterns and profiles, for a better prediction and prevention of different diseases and health crises in different individuals, towards advancements in personalized medicine.

The potential inter-disciplinary cross-fertilization between fields such as meteorology, human physiology and social psychology may be useful when developing methods to study and predict the influence of the environment on the development of the autonomic activity. The latest predictive methodology, combined with the traditional approaches based on the identification of linear and non-linear dynamic functions, are likely to be highly effective in supporting progress in the understanding, diagnosis and prediction of different physiological activity and diseases.

## 5. Conclusions and Future Directions

The results of this study about hemodynamic relationships between HR and BP across time supports the concept of ‘autonomic coordination’ reactive patterns with individual and tasks specificities. The finding also shows an interesting variety of time-based hemodynamic types reacting under the same tasks, which suggest a diversity of ‘autonomic coordination’ profiles across persons. The study discusses possible psychosocial factors related to this diversity, which may contribute to explain this large variety of hemodynamic responses. Further research integrating genetic and psychosocial predictors will open new avenues to advance in predictive and personalized medicine, using also novel methodologies from other different predictive disciplines through fruitful cross-fertilization. 

## Figures and Tables

**Table 1:** One-way repeated measures ANOVAs in heart rate, systolic blood pressure, diastolic blood pressure and mean blood pressure means between baseline, math task (*n* = 42) and rumination task (*n* = 34) conditions.

	Means (SD)			Wilks. Lambda	F	DF Hypot	DF Error	Sig.	Partial Eta Squared
	Baseline	Math Task	Rumination Task						
HR (bpm)	66.7 (17.9)	68.0 (17.6)	65.8 (18.1)	0.89	2.73	2	40	0.077	0.23
SBP (mmHg)	115.5 (31.1)	130.5 (32.7)	133.2 (34.9)	0.41	28.66	2	40	0.000	0.59
DBP (mmHg)	65.2 (19.3)	69.2 (18.9)	70.2 (22.0)	0.80	4.90	2	40	0.013	0.20
MBP (mmHg)	82.0 (23.0)	89.5 (23.1)	91.1 (25.3)	0.56	15.47	2	40	0.000	0.44

Abbreviations: HR—heart rate (bpm); SBP—systolic blood pressure DBP (mmHg); DBP—diastolic blood pressure (mmHg); MBP—mean blood pressure (mmHg).

**Table 2:** Contingency tables among types of individual heart rate—systolic blood pressure cross-correlations (CCs) profiles during the math and rumination tasks. Number of cases.

		Types of Profiles during Math Task						**Total**
		Positive CCs	Positive CCs under Negative Lags, Negative CCs under Positive Lags	Non-Significant	Several Cycles	Negative CCs	Negative CCs Under negative Lags, Positive CCs under Positive Lags.	
Types of profiles during rumination	Positive CCs	5	0	0	0	1	0	6
	Positive CCs under negative lags, negative CCs under positive lags	2	1	1	0	2	0	6
	Non-significant	1	0	0	0	0	0	1
	Several cycles	5	0	3	3	1	1	13
	Negative CCs	1	1	1	1	0	0	4
	Negative CCs under negative lags, positive CCs under positive lags	0	0	0	0	0	1	1
Total		14	2	5	4	4	2	31

## Data Availability

Not applicable.
